# Pre-Exposure to 50 Hz Magnetic Fields Modifies Menadione-Induced Genotoxic Effects in Human SH-SY5Y Neuroblastoma Cells

**DOI:** 10.1371/journal.pone.0018021

**Published:** 2011-03-23

**Authors:** Jukka Luukkonen, Anu Liimatainen, Anne Höytö, Jukka Juutilainen, Jonne Naarala

**Affiliations:** Department of Environmental Science, University of Eastern Finland, Kuopio, Finland; University of Massachusetts Medical School, United States of America

## Abstract

**Background:**

Extremely low frequency (ELF) magnetic fields (MF) are generated by power lines and various electric appliances. They have been classified as possibly carcinogenic by the International Agency for Research on Cancer, but a mechanistic explanation for carcinogenic effects is lacking. A previous study in our laboratory showed that pre-exposure to ELF MF altered cancer-relevant cellular responses (cell cycle arrest, apoptosis) to menadione-induced DNA damage, but it did not include endpoints measuring actual genetic damage. In the present study, we examined whether pre-exposure to ELF MF affects chemically induced DNA damage level, DNA repair rate, or micronucleus frequency in human SH-SY5Y neuroblastoma cells.

**Methodology/Principal Findings:**

Exposure to 50 Hz MF was conducted at 100 µT for 24 hours, followed by chemical exposure for 3 hours. The chemicals used for inducing DNA damage and subsequent micronucleus formation were menadione and methyl methanesulphonate (MMS). Pre-treatment with MF enhanced menadione-induced DNA damage, DNA repair rate, and micronucleus formation in human SH-SY5Y neuroblastoma cells. Although the results with MMS indicated similar effects, the differences were not statistically significant. No effects were observed after MF exposure alone.

**Conclusions:**

The results confirm our previous findings showing that pre-exposure to MFs as low as 100 µT alters cellular responses to menadione, and show that increased genotoxicity results from such interaction. The present findings also indicate that complementary data at several chronological points may be critical for understanding the MF effects on DNA damage, repair, and post-repair integrity of the genome.

## Introduction

Over the past thirty years, there has been scientific and public debate about the possible carcinogenic effects of extremely low frequency (ELF) magnetic fields (MFs), which are present wherever electricity is transmitted or used. The complexity of this issue was also recognized by the International Agency for Research on Cancer (IARC) in 2002, when it classified ELF magnetic fields as possibly carcinogenic to humans [1]. This evaluation was mainly based on rather consistent epidemiological findings showing an association between residential ELF MF exposure and childhood leukaemia. However, the underlying biophysical and biological mechanisms by which ELF MF might cause childhood leukemia, or carcinogenicity in general, are still unknown.

As the causal relationship between carcinogenicity and DNA damage is well established, possible genotoxic effects of ELF MF have been widely investigated. In these studies, MF alone has generally not been found to cause genetic damage [1], [2]. However, several studies with combined exposures have suggested that MF might enhance the effects of known DNA damaging agents [1], [2], [3]. Similarly, carcinogenicity studies in animals have not generally reported effects from MF alone, but studies with combined exposures have provided some evidence for cocarcinogenicity of MF [4], [5].

In a previous study [6], we found that pre-exposure to 50 Hz MF altered cellular responses (cell cycle arrest, apoptosis) to menadione-induced DNA damage. This effect was consistently seen in several independent experiments, while MF exposure after menadione treatment did not alter the responses. In addition, no effects were found after MF exposure alone or MF combined with UVB radiation. As menadione is a radical-inducing agent, these observations might indicate that MF affects free radical processes, as suggested by the radical pair mechanism [7]. Although the magnetic flux densities used (100–300 µT) were relatively high in comparison with the levels (below 1 µT) found to be associated with childhood leukemia in epidemiological studies, the finding is potentially important for understanding how MF exposure alters cellular responses to DNA-damaging agents. However, the study did not include measurements of actual genetic damage.

The aim of the present study was to evaluate the extent of chemically induced genetic damage in human SH-SY5Y neuroblastoma cells pre-exposed to 50 Hz MFs. For a comprehensive evaluation, we measured immediate DNA damage level using the Comet assay, DNA repair rate, micronucleus frequency as an indicator of the post-repair stability of the genome, and relative survival of the cells. Two different chemical agents were used for inducing DNA damage: menadione is a free radical-producing [8] and methyl methanesulphonate (MMS) is an alkylating agent [9]. In addition, all repetitions were performed without the presence of chemicals to study the role of MF exposure alone.

## Materials and Methods

### Reagents

The following reagents were used in this study: ethanol (Altia, Rajamäki, Finland); NaCl (FF Chemicals, Haukipudas, Finland); normal melting point agarose (FMC bioproducts, Rockland, USA); Dulbecco's modified Eagle medium (containing 4.5 g/l glucose), fetal bovine serum (FBS), 5000 unit/ml penicillin and 5000 µg/ml streptomycin (Gibco, Carlsbad, USA); NaOH (J.T.Baker, Deventer, Holland); ethylenedinitrilotetraacetic acid Titriplex III (Na_2_EDTA) (Merck KGaA, Darmstadt, Germany); SYTOX Green, fluorescent beads (6 µm) (Molecular Probes, Eugene, USA); sucrose (MP Biomedicals Inc., South Chillicothe, Ohio, USA); citric acid, sodium citrate (Riedel-de Haën, Seelze, Germany); ethidium bromide, IGEPAL, low melting point agarose, methyl methanesulphonate (MMS), RNase A, sodium lauroyl sarcosinate,Triton X-100, Trizma base (Tris) (Sigma-Aldrich, Steinheim, Germany).

### Cell culture

Human SH-SY5Y neuroblastoma cells (obtained from Dr. Sven Påhlman, University of Uppsala, Sweden) were cultured in Dulbecco's modified Eagle medium (containing 4.5 g/l glucose) supplemented with 10% heat-inactivated fetal bovine serum (FBS), 50 U/ml penicillin and 50 µg/ml streptomycin. Cell cultures were maintained in cell culture flasks (with a 75 cm^2^ cell culture area) (Nunc, Roskilde, Denmark) in a humidified incubator with 5% CO_2_ and a temperature of 37°C. Cells were harvested by 0.02% EDTA in Ca^2+^- and Mg^2+^-free phosphate buffer saline (PBS). Cells were plated in plastic 55 mm Petri dishes (Nunc, Roskilde, Denmark) for DNA damage level and repair rate experiments and in a 48-well plate (Nunc, Roskilde, Denmark) for micronuclei experiments. Cells were plated approximately 20 h prior to each exposure. The cell counts seeded per dish (2.0×10^6^) or well (10^5^) were selected from preliminary experiments to obtain subconfluent culture at the end of each exposure.

### MF exposure

The MF exposures were applied at a magnetic flux density of 100 µT for 24 hours. A comprehensive description of the exposure system has been published previously [6]. In brief, a pair of 340 mm×460 mm coils in a Helmholtz configuration (220 mm between the coils) generating a vertical magnetic field was housed inside a temperature-controlled cell culture incubator (Heraeus HERACell) with 5% CO_2_. Cell cultures were positioned at the center of the coil system to ensure a uniform magnetic field. Controls were maintained in an identical incubator without MF for the exposure period. Sinusoidal 50 Hz currents were generated by a Wavetek Waveform Generator model 75 (Wavetek, San Diego, CA, USA) and amplified by a Peavey M-3000 Power Amplifier (Peavey Electronics corp., Meridian, MS, USA). Magnetic flux density was monitored with a Holaday H1-3624 ELF Magnetic Field Meter and Holaday ELF Magnetic Field Sensor P/N 491017 (Holaday Industries, INC., Eden Prairie, MN, USA).

### Experimental protocol and chemicals

The cell cultures were exposed in four groups: (1) sham exposure (control), (2) MF exposure, (3) sham exposure + chemical treatment, and (4) pre-exposure to MF + chemical treatment. After the 24 h sham or MF exposure, the cells were either entered directly into the Comet assay or were treated with chemicals for 3 h and then assayed. In the micronucleus analyses, the 24-h sham and MF exposed cells were subjected to a 3-h incubation or 3-h chemical exposure respectively. Following this, cells were washed to remove the chemical and further cultivated in fresh medium for 69 hours (as the formation of micronuclei requires at least one cell cycle after the treatment).

Two different chemicals were used in the DNA damage level and repair rate assays, 20 µM menadione and 35 µg/ml methyl methanesulphonate (MMS). In addition, MMS at a concentration of 75 µg/ml was used as a positive control in the DNA damage level analyses to verify the performance of the Comet assay. The corresponding concentrations in the micronucleus assays were 0.1, 1, 10, 15, and 20 µM menadione and 10, 15, and 20 µg/ml MMS. Menadione is a semi-quinone that undergoes one-electron reduction in the mitochondrial respiratory chain, followed by one-electron transfer to molecular oxygen, producing O_2_^−•^, an essential oxygen radical [8]. The DNA-damaging effect of MMS is based on direct alkylation of DNA [9].

### Detection of DNA damage level and DNA repair rate

DNA damage level and repair rate were quantified by the Comet assay, also known as the single cell gel electrophoresis assay. In this assay, a sample of cells is mixed with agarose, spread onto a glass microscope slide and exposed to a lysis buffer, thus leaving only nuclei on the slides. During subsequent electrophoresis, the broken DNA fragments or damaged DNA migrate away from the nucleus and form a tail, resulting in an image that resembles a comet. The shape, size, and the fragment content of the tail reflect the extent of DNA damage. In the present study, DNA unwinding and electrophoresis were done under alkaline conditions (pH>13) in which the Comet assay detects DNA double-strand (DSB) and single-strand breaks (SSB). SSBs are associated with incomplete excision repair sites, DNA-DNA/DNA-protein cross-links and alkali labile sites (ALS) [10], [11].

To analyse DNA damage, cell culture dishes were treated with 3 ml of +37°C cell culture medium immediately after exposure(s). Samples were then placed on ice and cells were detached with 3 ml of 0.02% EDTA in PBS. After the cells were detached and samples were carefully suspended, 15 µl (∼1.5×10^3^ cells) of the cell suspension was embedded in 75 µl of 0.5% low melting point agarose. This mixture was carefully suspended and layered onto a microscope slide (pre-coated with a thin layer of 1% normal melting point agarose), immediately covered with a coverslip and kept on ice for 5 mins to solidify the agarose. After removal of the coverslips, the slides were immersed in a lysis solution (2.5 M NaCl, 100 mM Na_2_EDTA, 10 mM Tris, 1% sodium lauroyl sarcosinate, 1% Triton X-100, pH 10) and incubated for 1 h at +4°C in the dark. Following this, the slides were placed in a horizontal electrophoresis unit (Gibco-BRL, Horizon 20⋅25, Gaithersburg, USA) for 10 minutes, allowing DNA to unwind in the electrophoresis buffer (1 mM EDTA and 300 mM NaOH, pH>13, +4°C). The electrophoresis was run for 10 min at 24 V (0.66 V/cm) and 370 mA. After electrophoresis, the slides were neutralized (3×5 min) with Tris buffer (0.4 M, pH 7.5) and fixed in 96% ethanol for 1 min.

In the DNA repair rate analysis, cells were allowed to repair DNA damage, and were then subjected to analysis of DNA damage (the change from the initial DNA damage level may be interpreted as a measure of DNA repair). This was conducted by washing the cells with 3 ml of fresh medium and then allowing DNA repair to occur for 7.5 or 15 minutes in the cell culture incubator. The cells were then washed, detached, and assayed for DNA damage as described above.

For the analysis, slides were coded and stained with 20 µg/ml ethidium bromide. The analysis of 100 nuclei per slide was performed with a fluorescence microscope (Axio Imager.A1, Carl Zeiss, Göttingen, Germany) using the Comet assay IV (Perceptive Instruments, Haverhill, UK) image analysis software. Olive tail moment (OTM; a measure of tail length x a measure of DNA in the tail) was used as the parameter of DNA damage.

### Detection of Micronucleus frequency and relative survival

For measurement of micronuclei and relative survival, the cells were stained with ethidium monoazide bromide (EMA), photoactivated with visible light (light bulb), stained with SYTOX Green) and measured by flow cytometry [12]. This is a new method which is more objective than the traditional microscopic analysis and allows analysis of a much larger number of cells. The double-staining used allows differentiation of the nuclei of dead and living cells. Micronuclei are biological markers of genotoxicity, reflecting both chromosome loss and chromosome breakage [13].

After the exposure and incubation for 72 h, the medium was removed from the wells of the 48 well plates (2 wells per exposure group) and cell cultures were incubated on ice for 20 min. Following this, 150 µl of 8.5 µg/ml EMA-solution (+4°C) was added to each well and the cells were light activated on ice for 30 min. The light activation was performed under a table lamp with a distance of 15 cm between the plate (without a lid) and the light bulb. From this point forward, exposure of samples to light was minimized as much as possible. After the light activation, EMA-solution was removed from the wells, the cell cultures were washed once with 500 µl of 2% FBS in PBS (w/o Ca^2+^ and Mg^2+^, +4°C), 250 µl of Lysis 1-solution (0.3 µl IGEPAL/ml, 0.584 mg NaCl/ml, 0.5 mg RNase A/ml, 1 mg sodium citrate/ml, and 0.4 µM SYTOX Green in MilliQ-water, +4°C) was added and samples were incubated at +37°C for 1 h. After the incubation, 250 µl of Lysis 2-solution (15 mg citric acid/ml, 85.6 mg sucrose/ml, 0.4 µM SYTOX Green, and 1 drop of 6 µm fluorescent beads, +20°C) was added into each well and cell cultures were further incubated at room temperature (+20°C) for 30 min. At the end, cells were transferred into flow cytometer tubes and analyzed with a flow cytometer (Becton Dickinson FACScalibur, Becton Dickinson, San Jose, CA). Instrument settings and gating were done according to Bryce *et al.* [12]. Samples were protected from light and resuspended before analysis. Data were acquired and analyzed by CellQuest software v.3.3 (Becton Dickinson, San Jose, CA). A total of 2×10^5^ gated nuclei were scored per sample. As counting beads were present in every sample, the relative survival was calculated from nuclei to beads ratio.

### Statistical analysis

Statistical analysis of DNA damage level, DNA repair rate, micronucleus frequency, and relative survival was performed using linear mixed model analysis [14]. This analysis is an extension of classical one-way ANOVA and other general linear models, and more suitable than classical one-way ANOVA for complex exposure settings; in this case we needed to perform multiple-way comparisons (effects of multiple factors tested simultaneously), and there were multiple independent replicates. In the analysis, dish or replication pair was categorized as a random factor, while exposure to chemical(s), exposure to MF, measurement time (in DNA repair rate analysis), and their interactions, were considered as fixed effects. Pairwise comparisons between MF-exposed and sham-exposed samples were performed as post tests. Statistical analysis was performed by SPSS for Windows release 14.0.1 (SPSS Inc., Chicago, Illinois, USA) using raw or logarithm modified (DNA damage level and DNA repair rate) values. A p-value less than 0.05 was considered statistically significant.

## Results

### DNA damage level and repair rate

**Figure 1:**
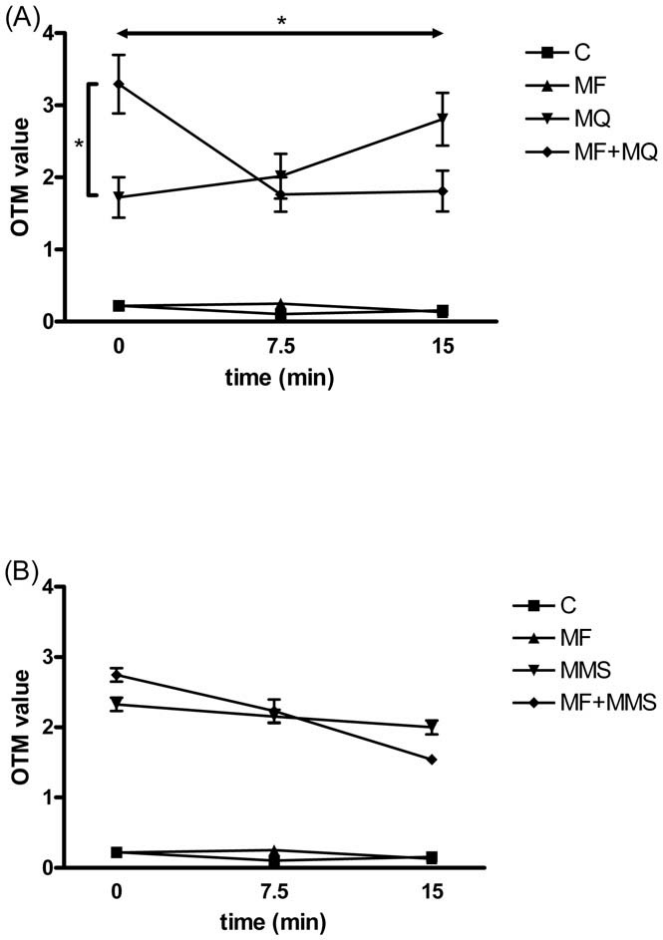
Effects of 50 Hz magnetic field (MF) and (A) 20 µM menadione (MQ) or (B) 35 µg/ml methyl methanesulphonate (MMS) on DNA damage level and DNA repair rate in human SH-SY5Y neuroblastoma cells. Both 20 µM menadione and 35 µg/ml MMS produced a statistically significant increase in DNA damage (p<0.01 and p<0.001, respectively). The symbol ↔ presents the overall change (DNA repair rate, 0 to 15 min) between menadione and MF + menadione exposed groups. Error bars represent SEM from three experiments with 100 nuclei analyzed in each experiment (thus, a total of 300 nuclei were analyzed), * =  p<0.05.

Figure 1 depicts the results of the Comet assays. In the DNA damage level assay (time point 0 min), both 20 µM menadione (Fig. 1A) and 35 µg/ml MMS (Fig. 1B) produced a statistically significant increase in DNA damage (p = 0.006 and p<0.001, respectively). In addition, DNA damage was increased in the MF + menadione exposed group compared to menadione exposure alone (p = 0.030; Fig. 1A). The DNA damage level was higher in the MF + MMS exposed cells compared to those exposed to MMS alone, but this difference was not statistically significant (Fig. 1B). MF exposure alone did not affect DNA damage level. The positive control (75 µg/ml MMS, OTM 24.54±0.62) significantly increased DNA damage level (p<0.001).

In the DNA repair rate analysis (time points 7.5 min and 15 min), DNA damage level continued to increase in the menadione-only exposed cells, whilst ongoing repair (reducing level of DNA damage) was observed in the MF + menadione exposed cells. The overall change (DNA repair rate, 0 to 15 min) was significantly (p = 0.039) different between the treatment groups, although the differences in DNA damage level at the individual time points (7.5 and 15 min) were not statistically significant. A frequency analysis was performed for further examination of the data (300 nuclei/each sample/time point). This analysis revealed that the number of heavily damaged nuclei (OTM value >10) increased (from 15 to 28) from time point 0 to 15 min in the menadione-only exposed samples, while this number decreased (from 33 to 20) in the MF + menadione exposed samples. It thus seems that pre-exposure to MF enhances DNA repair rate after exposure to menadione. The results from the experiments with MMS were qualitatively similar: DNA repair rate from 0 to 15 min was higher in the MF + MMS exposed group than in the MMS only exposed group, but this difference was not statistically significant. DNA damage was repaired statistically significantly (p = 0.013) in the MF + MMS exposed group, but not in the MMS only exposed group.

### Micronucleus frequency

**Figure 2:**
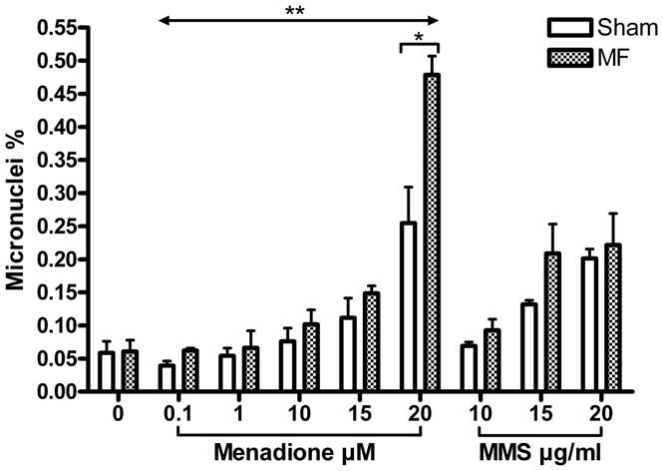
Effects of 50 Hz magnetic field (MF) and menadione (0.1, 1, 10, 15, and 20 µM) or methyl methanesulphonate (MMS; 10, 15, and 20 µg/ml) on micronucleus frequency in human SH-SY5Y neuroblastoma cells. The symbol ↔ represents MF + menadione exposed groups compared to groups exposed to menadione alone. Exposure to menadione (doses 15 and 20 µM) and MMS (15 and 20 µg/ml) caused statistically significant increase in micronucleus frequency (p<0.05, p<0.001, p<0.05 and p<0.01, respectively). Error bars represent SEM from three experiments, * =  p<0.05, and ** =  p<0.01.

Figure 2 shows the results of the micronucleus frequency assay. Both chemicals used in the experiments caused statistically significant increase in micronucleus frequency at the two highest doses (p = 0.038 and p<0.001 for the 15 and 20 µM doses of menadione respectively; p = 0.012 and p = 0.001 for the 15 and 20 µg/ml doses of MMS respectively), and these effects were dose-dependent.

Micronucleus frequency was significantly greater in the MF + menadione exposed groups than in the menadione only exposed groups (p = 0.007) when all menadione doses were included in the analysis. In separate tests for the different menadione doses, the difference was statistically significant at 20 µM (p = 0.047). MF exposure alone did not alter micronucleus frequency. Again, the results from the MMS experiments were qualitatively similar: the micronucleus frequencies were higher in MF + MMS exposed cells than in those exposed to MMS alone, but this difference was not statistically significant.

### Relative survival

**Figure 3:**
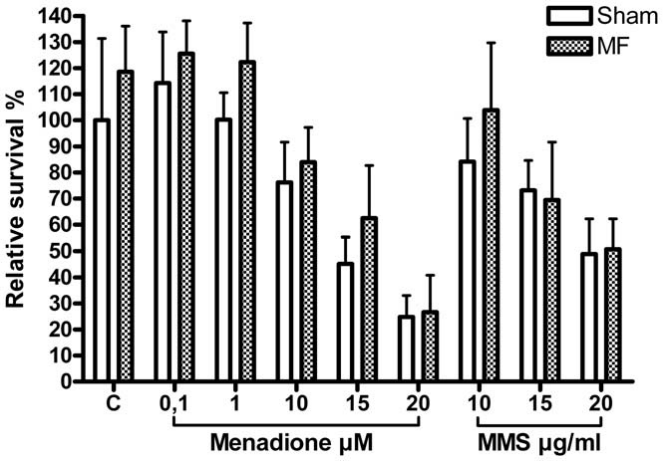
Effects of 50 Hz magnetic field (MF) and menadione (0.1, 1, 10, 15, and 20 µM) or methyl methanesulphonate (MMS; 10, 15, and 20 µg/ml) on relative survival in human SH-SY5Y neuroblastoma cells. Both menadione (15 and 20 µM) and MMS (20 µg/ml) resulted in statistically significantly decreases in relative survival (p<0.01, p<0.001, p<0.01, respectively). Error bars represent SEM from three experiments.

Figure 3 depicts the results of the relative survival assay. Exposure to high doses of menadione (15 and 20 µM) and MMS (20 µg/ml) resulted in statistically significant decreases in relative survival (p = 0.001, p<0.001, p = 0.002, respectively). Although relative survival was always higher in the MF + menadione exposed cells than in those exposed to menadione alone, this difference was not statistically significant in tests for individual menadione doses or in an overall test. No significant difference in relative survival was observed between the MMS only and MF + MMS exposed samples. MF exposure alone did not significantly affect the relative survival rate of the cells.

## Discussion

Our previous study showed that pre-exposure to ELF MF modified the cell cycle stages of menadione-exposed cells, suggesting both reduced apoptosis and increased cell cycle arrest [6]. Although these findings implicated alterations in cellular responses to DNA damage, the study did not include endpoints measuring actual genetic damage. In the present study, we evaluated several chronological steps of genetic effects, immediate DNA damage, DNA repair rate and post-repair stability of the genome. The results corroborate our previous findings that pre-exposure to MF alters cellular responses to menadione and show that the altered responses can also lead to enhanced genetic damage.

Pre-exposure to MF enhanced DNA damage level immediately after a subsequent menadione exposure (at time point 0 min). A number of previous studies have suggested that MF exposure might enhance the effects of known DNA damaging agents, but the evidence is still inadequate and in some cases conflicting [2].

Interestingly, pre-exposure to MF enhanced the repair rate of menadione-induced DNA damage. Previous findings concerning MF effects on DNA repair rate have generally been negative [15], [16], [17], apart from studies by Chow et al. who noted an increase in DNA repair efficiency [18] and Robison et al. who reported decreased DNA repair rate [19]. It is of interest to note that in all studies with negative findings [15], [16], [17], exposure to MF was after the initial DNA damaging treatment, while Robison et al. exposed cells to MF prior to the mutagen exposure [19]. Chow et al. did not use a mutagen [18]. Besides the sequence of the exposures, other experimental differences may also explain the differences in results, such as differences in magnetic flux density and frequency, model organism, and the DNA-damaging co-exposure agent used.

It is important to note that the increased DNA damage level (at time point 0 min) and enhanced DNA repair rate are not necessarily conflicting findings. A possible explanation for this finding might be that greater damage leads to different dynamics of the repair, which begins at an earlier time point. Alternatively, pre-exposure to MF might stimulate repair systems, but also open DNA structure for repair, thus making it more vulnerable to chemically induced DNA damage.

The enhanced DNA repair rate in MF-exposed cells resembles the so-called adaptive response. In the adaptive response, a prior exposure to mild stress causes improved resistance to a subsequent higher amount of stress. This phenomenon has been widely studied in cells exposed to ionizing radiation and chemical agents, but the observations of reduced chemically-induced apoptosis after a preceding MF exposure [6], [19] are also consistent with an adaptive response.

As mild oxidative stress generally leads to accelerated cell proliferation [8], the higher relative survival levels in the samples exposed to MF alone and the samples exposed to small menadione doses (0.1, 1 µM), although not significantly higher, are consistent with increased radical levels. However, the present research was not designed to evaluate either proliferation rate or radical levels and thus this hypothesis requires additional studies.

The mechanism of menadione-induced DNA damage may be of importance for the interpretation of the results. Menadione forms O_2_^−•^ radicals which at physiologically relevant levels are rather non-reactive with DNA [8], and it probably causes non-oxidative DNA damage [20] via activation of Ca^2+^-dependent nucleases [21]. This mechanism is closely linked to apoptosis-related DNA fragmentation [22], so the DNA repair results may partly reflect menadione-induced apoptosis (that continues during the repair assay) and suppression of such apoptosis by MF exposure. Suppression of apoptosis by MF pre-treatment is consistent with our previous data [6]. On the other hand, enhanced DNA repair is expected to increase cell survival and decrease apoptosis rate. Therefore, whatever the relationship between the observed DNA damage and apoptosis in the present results is, they are accordant with our previous findings [6].

The increased micronucleus levels in the MF + menadione-exposed cells suggests a defect in double-strand break (DSB) repair, as micronuclei are largely formed due to failure to correctly repair DSBs [23]. The level of micronuclei was consistently higher in the MF-exposed cells at all five menadione concentrations used. The micronucleus findings indicate that the enhanced rate of DNA damage removal in the MF-exposed cells did not lead to improved fidelity of DNA repair, consistent with previous findings showing that the rate and fidelity of DNA repair are not necessarily correlated [24], [25]. The findings of the present study actually show an inverse relationship between repair rate and repair fidelity. This observation (fast repair and increased micronuclei in the MF + menadione exposed cells) might reflect a causal relationship, if one assumes that it reflects dominance of the faster, but more error-prone, non-homologous end joining (NHEJ) repair pathway over the slower, but more precise, homologous recombination (HR) repair pathway [26]. There is evidence that the fast component of DSB rejoining and micronucleus yield are positively correlated [25].

The present results underline the importance of using several genotoxicity-related chronological steps instead of one separate time point. Increased DNA damage level was observed at the 0 min time point, but following time points showed no MF effects or even an opposite outcome (decreased damage). This indicates that differences in the time of measurement might partially explain conflicting results between studies of MF genotoxicity. Micronuclei, which are measured at a later point in time, can be considered as a measure of the integrity of the genome after DNA repair, and are therefore likely to be highly relevant for carcinogenicity. Micronucleus frequency has been found to be a biomarker of cancer predisposition *in vivo* [13].

In conclusion, the present results confirm our previous findings showing that MF pre-treatment at 100 µT alters cellular responses to menadione and contributes additional evidence that increased genotoxicity can result from such interaction. The present data also demonstrate that measuring several genotoxicity endpoints at different times after induced DNA damage may be critical for understanding MF effects on DNA damage, repair, and post-repair integrity of the genome.
